# Qualitative thematic analysis of the phenomenology of near-death experiences

**DOI:** 10.1371/journal.pone.0193001

**Published:** 2018-02-14

**Authors:** Helena Cassol, Benoît Pétré, Sophie Degrange, Charlotte Martial, Vanessa Charland-Verville, François Lallier, Isabelle Bragard, Michèle Guillaume, Steven Laureys

**Affiliations:** 1 Coma Science Group, GIGA-Consciousness, University and University Hospital of Liège, Liège, Belgium; 2 Department of Public Health, University of Liège, Liège, Belgium; 3 Family Medicine Department, University of Reims Champagne-Ardenne, Reims, France; The Chinese University of Hong Kong, HONG KONG

## Abstract

Near-death experiences (NDEs) refer to profound psychological events that can have an important impact on the experiencers’ (NDErs) lives. Previous studies have shown that NDEs memories are phenomenologically rich. In the present study, we therefore aimed to extract the common themes (referred to as “features” in the NDE literature) reported by NDErs by analyzing all the concepts stored in the narratives of their experiences. A qualitative thematic analysis has been carried out on 34 cardiac arrest survivors’ NDE narratives. Our results shed the light on the structure of the narratives by identifying 10 “time-bounded” themes which refer to isolated events encountered during the NDE and 1 “transversal” theme which characterizes the whole narrative and generally appears as a retrospective comment of self-reflection on the experience. The division of narratives into themes provides us with detailed information about the vocabulary used by NDErs to describe their experience. This established thematic method enables a rigorous description of the phenomenon, ensuring the inclusion of all self-reported manifestations of themes in narratives.

## Introduction

After facing a life-threatening situation, some people (so-called “near-death experiencers”; NDErs) report profound psychological experiences that may include features such as an intense feeling of peacefulness or joy, out-of-body experiences (OBEs), meeting a deceased relative or a spiritual figure, or seeing a brilliant light [1]. Such episodes, classically happening during an altered state of consciousness, have been termed “near-death experiences” (NDEs). Although there is still no single accepted definition of the phenomenon, a NDE can be referred to as “a profound psychological event including transcendental and mystical elements, typically occurring to individuals close to death or in situations of intense physical or emotional danger” [2].

Since the best seller “*Life After Life*” [1], testimonies of NDEs have been increasingly reported. This is not surprising, knowing that 6.3 to 23% of cardiac arrest survivors have experienced this phenomenon [3,4]. This important percentage reflects the fact that NDEs are widespread rather than sporadic events. Furthermore, some authors have shown that NDEs will generally change NDErs’ further attitudes and beliefs [5], and can even be distressing in case of negative NDEs (in some cases reported as “hellish experiences”) [6].

Many authors have contributed to the description of NDEs’ phenomenology. Moody [1] has delineated 15 recurrent characteristics notably including feelings of peacefulness and calm, hearing unusual voices, seeing a dark tunnel, being out of the body, meeting "spiritual entities" or seeing a panorama of one’s life events. A few years later, Ring [7] identified a 5-steps sequence that might tend to appear in the following order: 1) peace and well-being, 2) separation from the physical body, 3) entry in a dark area, 4) vision of a dazzling light, and finally 5) entry through the light into another realm. Later, other authors developed scales in order to rigorously investigate this phenomenon. Currently, the most used tool is the Greyson NDE scale [8]. This scale is a validated 16-item multiple-choice questionnaire used to quantify the intensity of the experience and to assess core content components of 16 NDE features [8,9].

Despite the significant number of people who reported to have had a NDE, there is still no theory that may account for all the characteristics classically described. Moreover, although it is now widely admitted that this experience is a physiological and psychological reality, a commonly accepted definition of the phenomenon is still lacking. Indeed, NDEs were initially named so because of their connection with death or fear of death [10]. More recently, however, similar experiences were reported in the absence of a physical or emotional threat (i.e., “NDE-like experiences”) [10–12]. Such experiences have been related, for instance, after meditation [13], syncope [14] and sleep [15]. Finally, Charland-Verville and collaborators [16] have shown that scores of NDE intensity (using self-reported responses on the Greyson NDE scale; [8]) do not differ between “real NDEs” experienced after a coma and “NDE-like” experiences occurring after non-life threatening events. Further systematic investigation is therefore required to better characterize these experiences and better describe their phenomenology.

Although a few studies have been conducting text analysis on NDEs narratives [17,18], reports of cardiac arrest survivors have never been, to our knowledge, analysed using a rigorous qualitative thematic method. Thematic analysis is “a method for identifying, analyzing and reporting patterns within data” [19] commonly used in qualitative research [20]. This method promotes the classification of the data into thematic categories as well as the examination of “all the cases in the study to make sure that all the manifestations of each theme have been accounted for and compared” [20]. Themes are patterns across data sets that are essential to a better description of a phenomenon. Therefore, thematic analysis can be used to develop taxonomies or classifications about a phenomenon [20]. Furthermore, well-established guidelines for applying and assessing qualitative methods are nowadays available [21,22], which have increased their use in medical disciplines [23].

The description of a NDE using closed scales can result in the overlooking of relevant features that might have been experienced by NDErs but that are not listed in NDE questionnaires. Therefore, this study aims to explore the interest of a qualitative approach, specifically thematic analysis, to better portray NDEs that follow a cardiac arrest based on self-reported narratives.

## Materials and methods

### Sample and recruitment

The study was approved by the ethics committee of the Faculty of Medicine of the University of Liège. NDErs were recruited via the websites, the appearances in local media and the publications of the International Association for Near-Death Studies (IANDS France) and the Coma Science Group (GIGA Research Center, University and University Hospital of Liège, Belgium). Participants who contacted us indicated their consent by signing a written consent form. They then completed questionnaires requesting socio-demographic information (gender and age at interview), their age when they experienced the NDE, the time elapsed since the NDE and if the NDE has occurred during a life threatening event.

In addition to these questions, participants were asked to write a detailed narrative of their experience. No limits regarding the number of pages were specified. The Greyson NDE scale [8] was then used to identify the presence of a NDE. This 16-item multiple-choice validated scale [8,9] allows the quantification of the intensity of the experience (i.e., total score ranging from 0 to 32) and enables a standardized identification of NDEs (i.e., cut-off score of 7). For each of the 16 items, the scores are arranged on an ordinal scale ranging from 0 to 2 (i.e., 0 = “not present,”1 = “mildly or ambiguously present,” and 2 = “definitively present”; [8,9]). NDEs being frequently reported after cardiac arrests, we included participants whose experience was secondary to a cardiac arrest and who met the accepted criteria of a NDE (i.e., Greyson NDE scale’s total score ≥ 7; [8]). **Table 1** shows the descriptive data of the study sample calculated using SAS version 9.3 for Windows statistical package).

**Table 1:** Greyson NDE scale total scores and descriptive data of the 34 NDErs (34 cardiac arrests; 11 females).

**Greyson NDE scale total score**	
Mean ± SD	13±5
Range	7–22
Reported time since NDE (in years)*	
Median	6
Interquartile range	4–10
**Age at NDE (in years)**	
Mean ± SD	49±13
Range	16–72
**Age at interview (in years)**	
Mean ± SD	56±12
Range	18–75

*This variable being not normally distributed, we used the median and the interquartile range. SD = Standard Deviation.

### Thematic analysis

According to the recommendations required for this method [19,24], an iterative step-by-step thematic analysis has been carried out on all anonymized 34 NDE narratives using NVivo software (version 9.2 for Windows). An inductive proceeding was chosen to analyze the data: themes were inductively defined from the raw data that were explored without any predetermined classification.

In the first step, two expert researchers in thematic analysis (SD and BP) read the narratives several times in order to familiarise themselves with the information.

In the second step, emergent themes were developed following a series of coding stages: first, open coding was used and initial codes were generated. Next, initial codes were grouped into categories according to their similarities.

In the third step, these categories were organized into themes. It involves combining codes into overarching themes that accurately depict the data. According to Braun and Clarke [19] “a theme captures something important about the data… and represents some level of patterned response or meaning within the data set”. This work led to a first analysis grid (i.e., list of themes).

In the fourth step, both experts independently extracted and classified all quotations (i.e., phrases or paragraphs) that corresponded to a theme of the analysis grid and preserved the quality of the writings as produced. To ensure the reliability of the coding and classification process, coding comparison query that enables to compare coding done by two experts in Nvivo, was performed by calculating a Cohen’s kappa coefficient. The kappa coefficient can range from -1 to +1 (+1 corresponding to a perfect concordance between the two experts). In the first instance, readers obtained a Cohen’s kappa coefficient of 0.46 demonstrating a moderate agreement [25]. That relatively poor result forced them to revise the analysis grid. According to the criteria of univocality and exclusivity (i.e., to ensure that themes are understandable in the same way by anyone and that quotations can be classified only in one single category), a new arrangement and a more precise definition of the themes have been proposed. Based on the revised grid (see **Table 2**), a new extraction and classification of quotations was achieved and led to a Cohen’s kappa coefficient of 0.73 between both readers corresponding to a substantial agreement [25].

**Table 2:** Coding structure of NDE narratives.

***Time-bounded themes***	*Light*: experiences in which the light is the main object. Associated subthemes: - Attractiveness - Description of the light - Source of light - Body sensation - Emotional feeling
	*Return*: transition between the "NDE" and "everyday life". Associated subthemes: - Opposition well-being/suffering - Attempt to relive the experience - No explanation - Order to come back
	*Meeting/encounter*: experience of an individual who encounters other beings (human or imaginary). Associated subthemes: - Focus on the bright environment around him/her or bright being - Message- content of the interaction - Typology of being met - Type of interaction - Body sensation - Emotional feeling
	*Hyperlucidity*: experience of omnipotence and extreme lucidity of the individual. Associated subthemes: - Outstanding intelligence, genius - Physical liberation - Links-unification-entirety-infinity - Omnipotence - Body sensation - Emotional feeling
	*Description of scenes*: experience of an individual describing the scene in which he is immersed. Associated subthemes: - Description of the place - Indescribable character of the place - Feeling of infinity - Body sensation - Emotional feeling
	*Darkness*: experience of an individual describing a dark, black, and deadly environment. Associated subthemes: - Description of the dark or black place - Contrast, black/light sequence - Person’s movement/environment or lack of movement - Sensory perception - Body sensation - Emotional feeling
	*Out-of-body experience*: experience of an individual who sees himself in an emergency situation from an observer’s perspective. Associated subthemes: - Awareness of being out of the body (state) - Experience of de-corporation (action of, way of getting out) - Visual description of the actual situation - High position, over flight - Attempt to communicate - Body sensation - Emotional feeling
	*Awareness of death*: state of people being aware that they are dying. Associated subthemes: No subcategory proposed
	*Life events*: experience of an individual who perceives different moments of his past or future life. Associated subthemes: - Actor/observer (notion of seeing or reliving the film of his/her life) - Content of the film (future projections vs. passed) - Imposed vs. free scrolling - Body sensation - Emotional feeling
	*Entrance in the NDE*: the moment when people enter the NDE. Associated subthemes: No subcategory proposed
***Transversal theme***	*Altered time perception*: depicts a change in NDErs’ perception of time. Associated subthemes: No subcategory proposed

The fifth step was made of a comprehensive analysis to examine the extent to which the themes contributed to an understanding of the data. For each theme, all the included quotations were synthesized to bring out the main ideas (i.e., a summary of the content provided by the participants).

The sixth step involved writing the report and led to a detailed description of the results. To ensure robustness, descriptive results were accompanied by quotations that illustrate that description. In addition, the number of NDErs who discussed the theme and its different characteristics was reported in order to summarize the results in a more illustrative and comprehensive form. A native English speaker translated the quotations included in the present article from French to English in order to preserve the NDErs’ subjective points of view.

### Validity and reliability of analysis

As recommended [22,24], several quality criteria were used to ensure the validity of the results and their interpretation: 1) triangulation: two complementary researchers (SD and BP) with different backgrounds (respectively MS in psychology and PhD in public health) performed the analysis; 2) researchers were experimented in qualitative research but novices in the NDE topic, which stimulates the exploration of new insights; 3) for each theme, the number of quotations were counted to get a more precise idea of their importance; 4) intercodage: a Cohen’s kappa coefficient was calculated; 5) theoretical validation: done by comparing the results with existing scientific data (see Discussion); 6) an iterative process was then performed. If a new code was introduced, all of the narratives were read again to ensure that the data extraction was complete and to verify that the initial classification was accurate.

## Results

### Extracted themes

The length of the narratives varied from 4 lines to 3 pages. The analysis conducted on the 34 narratives allowed us to distinguish 11 main themes, among which we identified 10 time-bounded themes and 1 transversal theme. A time-bounded theme refers to an event that is relatively isolated within narratives and only occurs during a part of the experience, whereas a transversal theme characterizes the whole experience and is not described as an isolated moment. Moreover, the transversal theme generally appeared in narratives as the result of a retrospective consideration and as a comment of self-reflection on the experience. Arbitrarily selected illustrative verbatim are given in **Table 3**. Themes are detailed in the paragraphs below.

**Table 3:** Arbitrarily selected examples of verbatim for each extracted theme.

NDE theme	Verbatim (gender; age at interview; reported time since NDE)
Light	“I moved forward. I was not walking. I felt attracted by the light and only my will was making me move forward.” (Male; 43 years old; 15 years) “Suddenly everything got spotless white. A very bright white that I had never seen before. As if there were a multitude of rays coming from this light. But it was not dazzling. It was glorious! Beings appeared and then disappeared, as if they were passing through the light.” (Female; 76 years old; 6 years) “The moment after, I was 'integrated' in an intense light that was so white that we cannot even imagine it, not enough words to describe that light. It is not dazzling, but it is extremely powerful, it is powerfully bright.” (Male; 61 years old; 20 years)
Return	“Quiet floating ride around the hospital and finally a meeting with a guy that sees me, hears me, ‘thinks’ me. He gives me the choice between two doors: this one, you go back home, but it will be difficult. OK, I go back, I cannot leave the children alone with their mother. Going back to my body is a horrible pain; I had simply forgotten what life was like, pain and 90% ignorance.” (Male; 50 years old; 15 years) “Everything disappeared all at once and I once again saw the doctors and the firemen taking care of me. The paramedic told me that I just had a cardiac arrest of 30 seconds. 30 seconds only, even though I have the impression that a very long time went by!” (Male; 52 years old; 4 years)
Meeting/encounter	“He talks to me. In fact, he does not talk to me: I am hearing his voice inside of me… I only remember one sentence, and I do not remember if he communicated anything else.” (Male; 61 years old; 20 years) “I was also seeing forms, of a human appearance but beheaded. They inspired me fear.” (Male; 44 years old; 7 years) “I saw my son who died at the age of 23.” (Female; 61 years old; 5 years)
Hyperlucidity	“I felt like I was a genius! I was thinking very fast, everything was working out well for me!” (Male; 64 years old; 9 years) “When my expansion was over, I was everywhere, I was everything at the same time, I was the sky, I was the ground, I was the trees and I felt the wind blowing in my leaves, I was the sea, and I was also my parents, my friends, people I had not met before but who, at that point, I knew because they were part of me. I was genuinely everything at the same time, everything was connected in one way or another.” (Female; 34 years old; 19 years) “My mind is lighter and quicker. It is free to think and to evolve without any restriction. I am still surprised about these infinite possibilities available to me as a mind, impossible to imagine or to realize with a physical body.” (Male; 61 years old; 20 years)
Description of scenes	“I found out I was on a kind of pirogue that followed the flow of a very black river. I was moving toward a bridge where beheaded beings were standing. They extricated other persons from the pirogues that passed under the bridge. They tore away their nails and they tortured them. It was really horrible.” (Male; 44 years old; 7 years) “From a moment to the next I found myself on the top of a hill, overlooking a huge place composed of forests of fir trees and flowers. I felt an indescribable wind, filled with happiness. The sky was filled with magnificent colors that I had never seen before. Clouds passed in the sky rapidly.” (Male; 44 years old; 7 years)
Darkness	“First, I remember being in a dark place, with no walls or rooms. I was wondering what I was doing there.” (Female; 76 years old; 6 years) “I heard a machine then nothing more, no more sound, nothing. I was in an absolute darkness.” (Female; 34 years old; 19 years)
Out-of-body experience	“It all starts with a very clear feeling: the certainty of leaving my body.” (Male; 61 years old; 20 years) “It is when I was transported to go down from the second floor on the stretcher that I had the strange feeling of seeing, from very high above the stairwell, that stretcher carried by four of my comrades that I was hearing as if they were very far. From that height, I could see everything.” (Male; 70 years old; 51 years)
Awareness of death	“I knew I was dead but I felt good, happy.” (Male; 56 years old; 6 years) “I am thinking: « Well, I am dead ». In fact, it is quite pleasant; I abandoned my life, my sick and emotionally troubled body for this marvelous state of well-being and peace.” (Male; 61 years old; 20 years)
Life events	“I do not know how it all began but I saw my life flashing before my eyes, essentially from the age of approximately 2 years old to 18. I had no notion of time. It seems like I was living whole scenes of my life over again. It was a real pleasure to live these happy moments over again, like when I was steeling the cherries from the trees when I was a child. I was feeling good. I did not want that moment to end.” (Male; 62 years old; 5 years) “I wandered for a while in what used to be my life, stopping, from time to time, on some events I had forgotten about or, by contrast, looking with delight at another scene that I had particularly enjoyed. Fragments deeply buried in my memory scrolled. Things that I had completely concealed from my conscious memory. I am surprised to find out about some scenes that seemed to be part of my life and that my memory has decided to overshadow. However, here they are! I am surprised to have done one thing or the other. All of this in a clear and sharp form.” (Male; 61 years old; 20 years)
Entrance in the NDE	“After a period of dark night, I found myself standing with no transition in a waiting room.” (Female; 51 years old; 11 years) “According to me, it seems that death comes relatively gently, at least in such a case. We open our eyes and we see tons of dancing lights. Even if I didn’t live the experience, the sensation of sliding in a bath of liquid nitrogen seems to be the best way to express what I felt. Then, simply, the pain stops.” (Male; 50 years old; 1 year)
Altered time perception*	“I am unable to tell how much time it lasted.” (Male; 65 years old; 6 years) “This impression of slow motion and of floating is still present within me, as if the notion of space and time had diluted.” (Male; 57 years old; 13 years)

*Transversal theme

#### Time-bounded themes

**Light**: Considering all narratives, 25 NDErs mentioned seeing a light. This light was attached to a feeling of attractiveness for 10 NDErs. 2 NDErs felt enveloped in this light. The description of the light comprised the following characteristics: intense (n = 16), white (n = 15), indescribable/unusual (n = 5), soft and diffuse (n = 3), not dazzling (n = 3), and yellow (n = 1). The physical sensations reported during this experience were an absence of body (n = 3) and an absence of pain (n = 1). NDErs expressed a feeling of happiness, serenity and tranquility (n = 15). The origin of the light was at the end of a tunnel or a corridor (n = 9), diffused (it came from everywhere; n = 7), or from an unknown origin (n = 1).

**Return**: 19 NDErs detailed the moment they got back from the NDE. 5 NDErs received a message that compelled them to get out of the experience. 4 of them reported being expelled or ejected from the experience. Getting back from the NDE was associated with an intense sleep (n = 2) or with a state of confusion (n = 2). 1 NDEr mentioned he had woken up after a period of dark night and 3 others characterized the return as brutal, without transition. 2 narratives included the idea of being brought back into the body. 2 NDErs did not remember how it happened. 1 NDEr attempted to live the experience again (which ended up in failure) and 12 NDErs mentioned an opposition between the feeling of well-being during the NDE and the problems they encountered when they got back to “everyday life”.

**Meeting/encounter**: A meeting with other beings (human or imaginary) was described in 15 narratives. The environment in which this meeting happened varied: a landscape (n = 1), a waiting queue (n = 1), an office (n = 1), in a light (n = 4), during a walk (n = 1), and on a river (n = 1). The message/the content of the interaction was mostly about getting back to life (n = 7). The type of interaction with the being also varied: unilateral message (only one being communicates towards the other; n = 4), telepathy (n = 4), or dialogue (n = 3). NDErs mainly saw their interlocutor (n = 11), however, others described the sensation of a presence (n = 2). The meeting happened with human beings that were unknown to the individual, relatives, family members (deceased (n = 8) or not (= 3)), and non-human beings. This experience was accompanied by a feeling of well-being (n = 4), an absence of pain (n = 3), fear (n = 2), unbearable sadness (n = 2), pain (n = 1), and confidence (n = 1).

**Hyperlucidity**: 14 NDErs reported a feeling of power and extreme lucidity. Hyperlucidity was associated with absolute clarity/understanding (n = 3), the feeling of being a genius (n = 2), clear and quick wit (n = 2), or exceptional intelligence (n = 1). This experience was in some cases accompanied by a physical release (n = 4). 5 NDErs described this experience as being accompanied by a sense of power and omniscience: direct control over the thoughts of others (n = 2), omnipotence (n = 2), or having an answer to everything (n = 1). 3 NDErs linked this supreme intelligence to the fact of being united with everything that surrounded them, to the global and universal character of this theme. This experience was associated with a feeling of well-being (n = 6), a lack of physical pain (n = 4), astonishment (n = 4), and an inability to describe the feeling (n = 1).

**Description of scenes**: 14 NDErs provided a detailed description of the setting in which they were immersed. 6 NDErs highlighted the indescribable aspect of the place (i.e., they showed difficulties in finding words). 4 NDErs evoked the idea of nature (e.g., vast meadow). This experience was accompanied by an intense feeling of well-being (n = 10), a feeling of infinity (n = 5), a lack of pain (n = 4), astonishment (n = 3), and fear (n = 1).

**Darkness**: 13 NDErs mentioned the idea of “black” or “dark”. They described a gloomy/dark environment with no objects or way out. For several NDErs (n = 7), this darkness occurred in contrast to or following a bright environment. More specifically, NDErs mentioned an absolute darkness (n = 7), a gloomy environment (n = 5), a gloomy tunnel (n = 2), a period of dark night (n = 1), and a waiting room with no walls (n = 1). 2 NDErs described an idea of movement (i.e., passing through a dark night). This experience was associated with the absence or the presence of sound (respectively n = 2 and n = 1), and the absence of sight (n = 2). This theme was linked to varied emotions: fear (n = 1), calmness (n = 1), and amazement (n = 1).

**Out-of-body experience**: 12 NDErs reported leaving their body. NDErs “saw” themselves (i.e., observer’s perspective). 4 NDErs evoked the awareness of being out of their bodies. A detailed visual description of the emergency situation was reported by 9 NDErs. 6 of them reported observing the scene from a higher position (positioned above). 3 of them reported having felt a real detachment of their body and 1 expressed the feeling of reintegrating his body. 2 NDErs said they wanted to communicate with the people they were observing, in vain. This experience was accompanied by an absence of pain (n = 3), thirst (n = 1), extreme cold (n = 1), and body perception (n = 1). The experience was also accompanied by a feeling of well-being (n = 7), amazement (n = 3), exasperation (n = 1), and rejection of the observed body (n = 1).

**Awareness of death**: 9 NDErs stated being aware that they were dying.

**Life events**: 8 NDErs out of 34 described a past or future life event. During these visions, NDErs perceived different moments of their past or future lives. Life was reviewed (n = 5) or relived (n = 2). The vision referred to the future life (n = 1) or, in the majority of cases, to the past life (n = 6). 3 NDErs stated that life passages comprised an alternation between happy and unhappy moments. These passages were imposed (n = 2) or selected (n = 1). This life review was associated with curiosity or surprise (n = 3), happiness (n = 2), difficulties in reviewing (n = 2), or with a feeling of indifference (n = 1).

**Entrance in the NDE**: 6 NDErs detailed the moment they entered the NDE. For 3 of them, the entrance was progressive and soft. For 2 others, the entrance followed a period of dark night. Another NDEr wrote he did not know how it all started.

#### Transversal theme

**Altered time perception**: 16 NDErs mentioned a change in the perception of time during their experience. 8 NDErs expressed a total loss of time marker. 6 NDErs reported an impression of slow motion or the feeling that time had stopped. Finally, 3 NDErs reported an unusual and ineffable perception of time, and therefore described it in an uncommon way (e.g., “integrated time”).

## Discussion

Since the past three decades, considerable work has been undertaken to describe NDEs in sufficient details, however, most studies have been using closed NDE questionnaires in order to identify the presence of an NDE and assess this phenomenon (e.g., [16,26]). Yet, previous studies [26–29] have shown that NDEs memories contained more characteristics than other memories of imagined and real events, which highlights the fact that we possess very rich and detailed narratives of these experiences. We therefore aimed to examine all the details stored in NDEs narratives using a qualitative thematic analysis.

One of the major contributions of our study is that it sheds a different light on the structure of the narratives by identifying 1 “transversal” theme and 10 “time-bounded” themes. More specifically, “transversal” themes characterize the whole narrative and do not correspond to a specific moment of the experience. Moreover, the transversal theme (i.e., “altered time perception” in this particular case) is generally addressed retrospectively by NDErs as they reflect upon their experience. On the contrary, “time-bounded” themes have more limited time duration and are generally described as clear isolated events (e.g., “OBEs”).

The originality of our approach also resides in the design of the study intended to provide new insights regarding the extracted themes. Firstly, the aetiology group of our participants was not revealed to the readers until the end of the analysis, and secondly, they were not experts in the field of NDEs. Obviously, NDEs are regularly discussed in the lay literature and the readers may not have been fully blinded, however, we wanted to enhance methodological rigor and reduce measurement biases by limiting any substantial influence of existing literature and preconceived notions on the processing of the narratives. For example, researchers’ knowledge of existing quantitative scales (e.g., Greyson NDE scale; [8]) could have had an incidence on the extraction process of the themes. By proceeding on this basis, we notably aimed at highlighting themes which might have been left aside in previous studies.

In the end, we identified the “altered time perception” as a transversal theme. This loss of time marker has already been reported in the NDE literature as a defining NDE feature (e.g., [5,8]). Concomitantly, 10 time-bounded themes have also emerged from our analysis, among which 7 are similar, or even identical, to the features that are described in the Greyson NDE scale [8] and the WCEI [7]: the vision of a “light”, a “meeting”, “hyper-lucidity”, “darkness”, “OBEs”, “life events”, and “awareness of death”. It is worth noting that some of the themes that have emerged from our qualitative thematic analysis include several features of the Greyson NDE scale (e.g., “life events” include “life reviews” as well as “precognitions”). Besides, some of the components we have identified are less specific. For instance, we identified the theme “light” whereas the Greyson NDE scale focuses on a “brilliant”, “unusual” or “mystical” light.

In parallel, readers identified 3 additional themes that partly overlap with the features identified by Greyson [8] and Ring [7]–namely, “entrance in the NDE”, “return” and “description of scenes”. These themes seem however to be described differently and to integrate complementary information and details. Indeed, for the theme “entrance in the NDE” some NDErs remembered and spontaneously detailed the way their NDE began, abruptly or progressively. As regard to the other two themes, “return” and “description of scenes”, it must be noted that they somehow overlap with existing features assessed by the widely used Greyson-NDE scale. If we consider, in the first instance, the theme “return”, we can establish the link with the concept of “border” raised by the Greyson NDE-scale. Indeed, the theme encompasses the decision to get back to life and the fact that some NDErs felt like they were sent back against their will. Yet, the theme “return” is broader than Greyson’s feature as it also comprises the way NDErs felt when returning to “usual awareness” and the substantial gap that exists between the emotions experienced during the NDE and during everyday life. Indeed, part of the NDErs emphasized the opposition between the feeling of well-being during the NDE and the problems encountered or the pain they felt when they got back from their NDE. It is our view that this latter finding could at least partially explain some of the beneficial consequences of NDEs such as a reduced fear of death [30]. The way NDErs came back from their NDE [1] and how resentful and frustrated they could feel about it [4] have already been brought to light by some authors, and we therefore believe that this issue deserves careful consideration in the future. Second, the theme “description of scenes” could lead one to think to the unearthly world reflected in the Greyson NDE-scale. Nonetheless, this theme not only includes unfamiliar and mystical places but also comprises the description of the setting in which NDErs found themselves during their NDEs (e.g., an operating room or the scene of the incident). Interestingly, even though some of the places they saw were portrayed as ineffable and indescribable, participants endeavored to detail them.

To our knowledge, no NDE questionnaire formally investigates those 3 themes and we believe that further work should be done to collect them more systematically. Furthermore, it could be interesting to explore the way they are described in narratives relating NDEs of other aetiologies.

Overall, these results corroborate the content of existing tools such as the Greyson NDE-scale [8], but also highlight new aspects of NDEs that could be further investigated in future studies. This recurrence in the extracted themes/features supports the view of authors such as Facco and collaborators [31] who suggest that NDE testimonies from all around the world show sufficient commonality to consider NDEs as “universal human experiences”.

In addition to outlining the structure of narratives, text analyses explore the context of reported themes and provide detailed descriptions of NDEs. In our sample, the “light”, usually qualified as “brilliant”, “unusual” or “mystical” in the NDE literature (e.g., [8]), is also described as “soft”, “diffuse” and “white”–interestingly, a majority the NDErs who saw the light depicted a white light. Besides, a majority of NDErs evoked a variety of strong feelings to describe most of the extracted themes and depicted a wide range of emotional states. Indeed, NDErs not only mentioned feelings of peace or joy but also reported feelings of astonishment, amazement, surprise and fear to describe the identified themes. In addition, it is worth noting that negative feelings such as fear are also comprised in positive NDEs. This wide range of emotions partly explains the fact that the feature “feelings of peace, pleasantness or joy”, consistently reported in other studies on cardiac arrest survivors (e.g., [3,16,32]), has not been high-lightened as a key element following our analysis. Even though pleasant feelings were frequently reported, readers considered that their identification as a key theme would have masked the diversity of the emotions felt by NDErs, especially negative ones. In addition, readers judged that positive feelings, such as pleasantness or joy, did not appear as a theme on their own but rather characterized some of the other themes that have been identified by the readers. As a matter of fact, “light”, “hyper-lucidity”, “OBEs” and “awareness of death” are generally associated with positive feelings (e.g., well-being, happiness, serenity or amazement). On the contrary, “meeting”, “darkness”, “life events”, “description of scenes” and “return” are related to conflicting emotions. Finally, “entering the NDE” and “altered time perception” rarely have an emotional value. In most cases, NDErs seem to use a wide range of qualifiers to describe their experience, which makes each narrative unique. In a nutshell, it has to be underlined that the “keyness” of a theme in qualitative analysis does not fundamentally lie on quantifiable measures but rather depends on the researcher’s judgement based on the content of the accounts [19].

Existing questionnaires, such as the Greyson NDE scale, allowed researchers to gather data and identify NDErs. Nonetheless, these tools do not offer the possibility to distinguish between NDEs of different aetiologies, or between classical NDEs and “NDE-like” experiences. In addition to the use of those scales, we believe that thematic analysis, combined with other types of qualitative analysis methods such as discourse analysis [33], should help to explore this issue further. Yet, qualitative analysis methods require good quality narratives and appropriate data collection. In this sense, it would be interesting to have a more systematic approach for data collection in order to obtain more detailed narratives. Semi-structured interviews are recommended in such a framework, requiring well-constructed interview guides. Moreover, it could be of a great interest to look into the meanings attached to such experiences and to analyze how NDErs reflect upon NDEs and their consequences on their lives. Besides, out of concern for methodological rigour, only narratives that scored 7 or more on the Greyson NDE scale were included in our study sample. Still, it would be interesting to carry out analyses on the narratives of all the NDErs who have contacted us after a cardiac arrest, without taking any cut-off score into account.

Finally, some issues remain to be addressed. First, the sample size in the present study is limited. Qualitative research is, however, very intensive and time consuming, which makes analysis of large samples impractical. Indeed, sample size in qualitative research is frequently much smaller than in quantitative study and generally does not exceed 50 participants [34]. Besides, given the preliminary use of the presented methodology, we decided in the first instance to restrict the analysis to the narratives of people who had lived comparable life-threatening situations, namely a cardiac arrest. As already mentioned above, it would however be of a great interest to invest in further analysis of reports depicting other types of experiences such as “NDEs-like” or close brushes with death that did not lead to a NDE. Second, because NDErs voluntarily contacted us, our sample might suffer from a self-selection bias. Due to the mystical connotation of these experiences, and because they may be perceived as negative and upsetting, some people might feel reluctant to share these events. Lastly, narratives were collected retrospectively and time elapsed since the NDE varies which could represent a source of potential bias. Indeed, one can hypothesize that NDErs’ narratives may have been tainted by descriptions of the phenomenon in medias and in lay literature. Consistent with this hypothesis, Charland-Verville and collaborators [16] pointed out that some NDE features seemed to be more frequent in retrospective designs. We underline, however, that Greyson [35] had highlighted, prior to that, the consistency of NDE memories over a period of two decades. Given this potential bias and the ineffability of the experience, it would be interesting to collect NDE accounts and look into the terms used by NDErs when recovering from their coma and depicting the experience for the first time. These observations and potential biases call for further studies to analyze prospective trials using thematic analysis method.

## Conclusion

Given the increasing number of NDEs testimonies and the impact that these experiences may have on peoples’ lives [5], it appears crucial to better describe the phenomenon. In this study, a qualitative thematic analysis was used to detail the rich phenomenology of these experiences. We were able to extract 11 themes. Among those, we notably identified 3 themes that partly overlap with features described in closed NDE questionnaires, but which seem to integrate complementary details. The division of narratives into themes gives a fuller overview about the way different features are combined and sheds a light on how these experiences, usually reported as being ineffable, are described by NDErs. Nevertheless, further work should be done to develop better tools to rigorously collect narratives, such as semi-structured interviews, in order to obtain standardized and detailed narratives.
